# Supplementary material for: Evidence supported by Mendelian randomization: impact on inflammatory factors in knee osteoarthritis
Source: Front Med (Lausanne). 2024 May 28;11:1382836. doi: 10.3389/fmed.2024.1382836 (PMC11165061; doi:10.3389/fmed.2024.1382836)
Supplement: Supplementary file 1 [file Data_Sheet_1.ZIP › data/GCST90274840_5e6/840.leaveoneout.pdf]

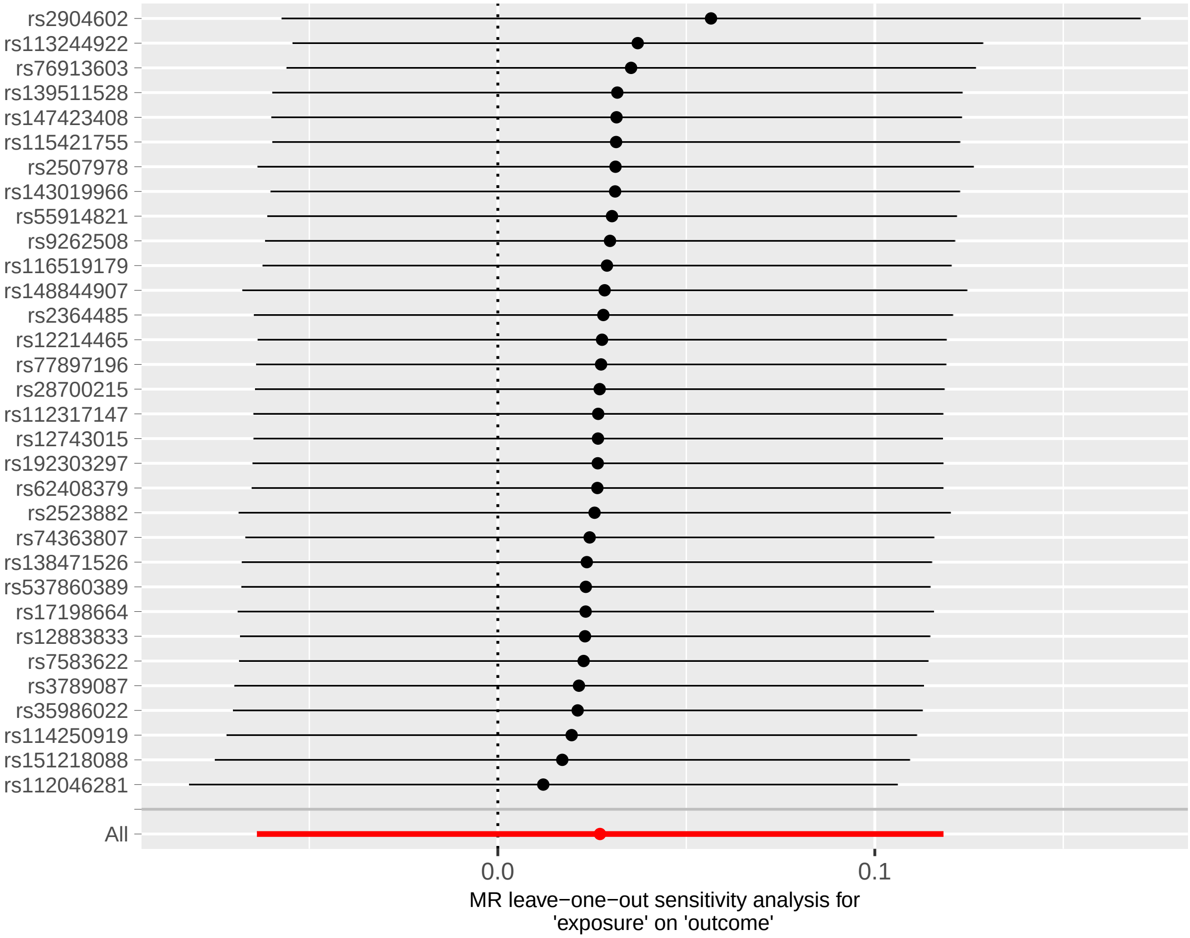
rs2904602
rs113244922
rs76913603
rs139511528
rs147423408
rs115421755
rs2507978
rs143019966
rs55914821
rs9262508
rs116519179
rs148844907
rs2364485
rs12214465
rs77897196
rs28700215
rs112317147
rs12743015
rs192303297
rs62408379
rs2523882
rs74363807
rs138471526
rs537860389
rs17198664
rs12883833
rs7583622
rs3789087
rs35986022
rs114250919
rs151218088
rs112046281
All
0.0
0.1
MR leave−one−out sensitivity analysis for
'exposure' on 'outcome'
